# Eating disorders and psychosis: a case report and review of the literature

**DOI:** 10.1192/bjo.2021.117

**Published:** 2021-06-18

**Authors:** Defne Flora Goy, Erdem Efe, Özge Şahmelikoğlu, Ümit Haluk Yeşilkaya

**Affiliations:** Bakirkoy Research & Training Hospital for Psychiatry, Neurology and Neurosurgery

## Abstract

**Aims:**

Despite evidence from case series, the comorbidity of eating disorders (ED) with psychosis is a challenging field to which little attention has been paid. There is no consistent sequence in the co-occurrence of the two conditions-eating disorders sometimes precede, and sometimes follow the onset of psychosis. In this case report, we present a 25-year-old female patient suffering from recurrent episodes of binge eating and inappropriate compensatory purging behaviours with psychotic components to discuss the co-occurrence of these conditions in the light of the literature.

**Method:**

Our patient suffered from sleep disturbances, homicidal thoughts, self-induced vomiting worsened in one year. Psychiatric examination revealed psychotic symptoms such as blunted affect, persecutory delusions, and delusions of appeal and justification. In our inpatient psychiatry clinic, she was treated with olanzapine 20 milligrams(mg) and quetiapine 500 mg per day.

**Result:**

Psychotic episodes occur in 10–15% of eating disorder patients. The prevalence of primary psychotic diseases like schizophrenia and schizoaffective disorders in eating disorder patients appears to be comparable to that in the general population. An ED can be the early sign of an impending psychosis, or psychotic symptoms can signal the beginning of an ED. The advent of the psychosis, and sometimes the treatment of the psychosis can cure the eating disorder, but it can sometimes aggravate it. The case presented illustrates the difficulties in managing a patient with a comorbid eating disorder and psychosis. To ensure a rigorous assessment of both psychotic and eating disorder symptoms, the focus should be on the pattern of appearance or emergence of symptoms, their phenomenology, clinical and family background of the patient, and clinical status on follow-up.

**Conclusion:**

The comorbidity between eating disorders and psychosis is infrequent and raises several conceptual and methodological questions. Epidemiological and family studies show that there is no more significant association between psychosis and ED, although these results are somehow limited by the lack of rigorous data regarding ED.

